# Identification of *Burkholderia gladioli* pv. *cocovenenans* in Black Fungus and Efficient Recognition of Bongkrekic Acid and Toxoflavin Producing Phenotype by Back Propagation Neural Network

**DOI:** 10.3390/foods13020351

**Published:** 2024-01-22

**Authors:** Chen Niu, Xiying Song, Jin Hao, Mincheng Zhao, Yahong Yuan, Jingyan Liu, Tianli Yue

**Affiliations:** 1 College of Food Science and Technology, Northwest University, Xi’an 710069, China; niuchen@nwu.edu.cn (C.N.); songxiying@stumail.nwu.edu.cn (X.S.); haojjin@stumail.nwu.edu.cn (J.H.); yyh324@tom.com (Y.Y.);; 2 The 20th Research Institute of CETC, Xi’an 710068, China

**Keywords:** black fungus, *B. gladioli*, bongkrekic acid, toxoflavin, MLST, neutral network

## Abstract

*Burkholderia gladioli* pv. *cocovenenans* is a serious safety issue in black fungus due to the deadly toxin, bongkrekic acid. This has triggered the demand for an efficient toxigenic phenotype recognition method. The objective of this study is to develop an efficient method for the recognition of toxin-producing *B. gladioli* strains. The potential of multilocus sequence typing and a back propagation neural network for the recognition of toxigenic *B. cocovenenans* was explored for the first time. The virulent strains were isolated from a black fungus cultivation environment in Qinba Mountain area, Shaanxi, China. A comprehensive evaluation of toxigenic capability of 26 isolates were conducted using Ultra Performance Liquid Chromatography for determination of bongkrekic acid and toxoflavin production in different culturing conditions and foods. The isolates produced bongkrekic acid in the range of 0.05–6.24 mg/L in black fungus and a highly toxin-producing strain generated 201.86 mg/L bongkrekic acid and 45.26 mg/L toxoflavin in co-cultivation with *Rhizopus oryzae* on PDA medium. Multilocus sequence typing phylogeny (MLST) analysis showed that housekeeping gene sequences have a certain relationship with a strain toxigenic phenotype. We developed a well-trained, back-propagation neutral network for prediction of toxigenic phenotype in *B. gladioli* based on MLST sequences with an accuracy of 100% in the training set and an accuracy of 86.7% in external test set strains. The BP neutral network offers a highly efficient approach to predict toxigenic phenotype of strains and contributes to hazard detection and safety surveillance.

## 1. Introduction

Black fungus (*Auricularia auricula-judae*) is one of the four cultivated edible fungi in the world, and it has been cultivated in China for more than one thousand years [1]. It is not only rich in carbohydrates, protein, fatty acids, vitamins, trace elements, and other essential nutrients [2], but also has in vivo and in vitro anti-oxidation [3], anti-tumor [4], hypolipidemic [5], immunomodulation, and other biological activities [6]. According to the reports by the China Edible Fungi Association, the annual output of dried black fungus in China was 6.90–8.29 megatons from 2016 to 2020 [7], and black fungus planting is a pillar industry in the Qinba Mountain area. However, one of the most dangerous food safety hazards harassing black fungus is *Burkholderia gladioli* pv. *cocovenenans.* It has been confirmed that the fatal toxicity associated with *B. gladioli* pv. *cocovenenans* comes from the two secondary metabolites toxoflavin [8] and bongkrekic acid [9] produced by the strain. Toxoflavin inhibits the respiratory chain and produces peroxide, which causes damage to vascular smooth muscle and organs [10,11]. Another toxin, bongkrekic acid is a mitochondrial toxin, which affects the normal metabolism of cells by inhibiting mitochondrial adenine nucleotide translocatase. The clinical manifestations of the poisoned patients are abdominal pain, vomiting, diarrhea, fatigue, irritability, shock, multiple organ failure, diffuse cell dysfunction, and death [12]. Food poisoning incidents caused by this bacterium poisoned more than 9000 people and led to more than 1000 deaths in China [13]. Moreover, its influence has spread abroad and made it a global safety issue in numerous foods [12,14], including coconut, Pompeii wine, fermented cereal, potato products, and tremella.

Although food poisoning incidents caused by *B. gladioli* pv. *cocovenenans* in black fungus and other food categories have been reported, *B. gladioli* pv. *cocovenenans* contamination still remains unclear in terms of the black fungus industry and toxin production in different food categories. Therefore, it is of great significance to unravel *B. gladioli* pv. *cocovenenans* contamination on black fungus.

The traditional method for recognition of *B. gladioli* pv. *cocovenenans* is differential medium cultivation and an update method is 16S rDNA sequencing [15]. However, these methods lack accuracy and can only discriminate on the species level but not the virulence pathovar level. The toxigenic phenotype can only be identified by toxigenic cultivation and subsequent toxin detection, which is tedious and cumbersome. The cultivation time to produce secondary metabolites greatly hinders the detection speed for food safety surveillance by the government, product quality control by food companies, and pathogen identification by researchers and health sectors. There is an urgent need to establish a new method for toxigenic phenotype recognition.

Multilocus sequence typing (MLST) assigns an arbitrary and unique allele number for each sequence in housekeeping gene loci and make designations for the alleles and deposited them in the MLST database [16]. The database deposited 108 allele sequences under *Burkholderia gladioli* [17] and provided sequence type (STs) for comparing *Burkholderia gladioli* isolates and studying biological population structure globally.

Deep learning is a sub-field of artificial intelligence and has exerted a great influence on a global scale. It mainly trains and learns the key conceptual elements of big data through in-depth training of neural network calculation methods, so as to enable machines to successfully identify and classify data [18]. A back propagation neural network model is a multi-layer feed forward neural network trained according to the error back propagation algorithm, which was put forward by a group of scientists led by Rumelhart and McClelland in 1986 [19]. The BP neural network model is an emerging method for food safety. By utilizing back propagation, the weights and thresholds of the network are continuously adapted to minimize the sum of squared errors, enabling data classification and recognition [20]. The BP neural network model is more driven by the data themselves, rather than the processing rules as the core compared with traditional data processing methods, so it can identify the data rules that people cannot [21]. For instance, a particle swarm optimization–BP neural network (PSO-BP) was put forward to build a prediction model of the number of *Escherichia coli* in Dai specialty snacks [22]. Also, a simple and accurate system for real-time identification of fish freshness has been developed based on the BP neural network trained with the marked images of anthocyanins in red cabbage [23].

In this study, the *B. gladioli* pv. *cocovenenans* strains in the environment of black fungus planting in Qinba Mountain area were isolated. The production of bongkrekic acid and toxoflavin toxins by isolates and standard strains in different foods was evaluated. A BP neural network model based on MLST sequences were established to predict the toxigenic capability of *B. gladioli* pv. *cocovenenans* and make predictive associations between phenotype and genotype. The BP neural network-based recognition method is rapid, independent of complex instruments and follow-up analysis, and does not need tedious physical and chemical experiments to detect pathogens.

## 2. Materials and Methods

### 2.1. Strain Isolation and Molecular Identification

In this study, black fungus and environmental samples were collected at the planting base (109.14 °E, 33.69 °N) of Zhashui Zhongbo Black Fungus Science and Technology Development Co., Ltd. in Zhashui County, Shangluo City, Shaanxi Province, referring to GB4789.29-2020 with some modification. Briefly, after GVC enrichment, broth enrichment (GVC, 6.0 g/L of potato leaching powder of, 20.0 g/L of glucose, 0.01 g/L of gentian violet, 0.02 g/L of chloramphenicol), modified potato dextrose agar coating cultivation (mPDA, 6.0 g/L of potato leaching powder, 20.0 g/L of glucose, 0.01 g/L of gentian violet, 0.02 g/L of chloramphenicol, 15.0 g/L of agar), PCFA medium streaking cultivation (PCFA, 1.0 g/L of NH_4_Cl, 1.14 g/L of KH_2_PO_4_, 0.7 g/L of Na_2_HPO_4_, 4.0 g/L of NaCl, 0.005 g/L of CaCl_2_, 0.2 g/L of MgSO_4_·7H_2_O, 0.0005 g/L of FeSO_4_·7H_2_O, 0.01 g/L of cystine, 0.05 g/L of glucose, 15.0 g/L of dulcitol, 12.0 g/L of agar), Gram staining, and egg yolk agar base cultivation (15.0 g/L of peptone, 3.0 g/L of beef powder, 5.0 g/L of NaCl, 15.0 g/L of agar, 10.0 g/L of glucose), pure culture of the suspected strain was obtained and stored in 30% glycerol at −80 °C for subsequent research.

The genomes of the suspected strains were extracted, 16S rDNA sequences were amplified by primers of 1492R (5′-GGTTACCTTGTTACGACTT-3′) and 27F (5′-AGAGTTTGATCCTGGCTCAG-3′) [15], and then the PCR product was purified by gel cutting and sequencing.

Two-way sequencing was adopted and the obtained sequences were spliced. Finally, the 16S rDNA sequences of the suspected strains were obtained. The sequences were searched by the Basic Local Alignment Search Tool (BLAST) in NCBI database to identify the species and genus of the strain (Supplementary Table S1).

### 2.2. Toxigenic Phenotyping

#### 2.2.1. Single-Strain Culture on PDA Medium

Twenty-six isolates and three standard strains Co14, DSM 11318, and ATCC 10248 were activated in liquid PDA medium for 48 h. Fifty milliliters of bacterial culture was centrifuged to remove the supernatant, and 500 µL of sterile saline solution was used to resuspend the bacteria. Then, the resuspended bacterial culture was inoculated onto PDA semisolid medium pre-covered with sterile cellophane, and cultured at 26 ± 1 °C for 5 d. Then, the PDA semisolid culture medium was transferred to a 50 mL tube after taking off the cellophane layer coated with bacteria, and sterilized at 100 °C for 30 min, cooled at room temperature, and frozen in a refrigerator at −20 °C overnight. These crude toxins had three replicate samples.

#### 2.2.2. Co-Culture with Rhizopus Oryzae on PDA Medium

Twenty-six isolates and three standard strains Co14, DSM 11318, and ATCC 10248 were activated in liquid PDA medium for 48 h. The co-culture method was modified from reference [24]. Briefly, the bacterial culture was inoculated on one side of a semisolid PDA plate pre-covered with sterile cellophane by a sterile cotton swab and cultured at 26 ± 1 °C for 2 d. *Rhizopus oryzae* was streaking inoculated on the other side of the PDA plate and the co-culture with isolates at 28 ± 1 °C for 7 d. Finally, the cellophane with bacteria was removed and the remaining medium was transferred to a 50 mL tube, sterilized at 100 °C for 30 min, cooled at room temperature, and frozen at −20 °C overnight. The crude toxin had three replicate samples.

#### 2.2.3. Cultivation on Soaked Black Fungus

One hundred microliters of culture of the activated 26 isolates and 3 standard strains were added into a soaked black fungus system (water 50 mL and fungus 2.0 g), respectively, at about 10^6^ CFU/mL inoculum, and with a subsequent cultivation at 26 ± 1 °C for 72 h. The samples were used for determination of bongkrekic acid and toxoflavin.

#### 2.2.4. Cultivation on Different Foods

The strains NC18, NC22, Co14, and DSM 11318 were activated to about 10^7^ CFU/mL. Subsequently, 100 µL of bacterial culture was inoculated into sterile fresh black fungus, tremella, chow fun, and rice noodle (each contains 30 mL of sterile water and 20.0 g of food), respectively. They were then cultured at 26 ± 1 °C for 3 d for determination of bongkrekic acid and toxoflavin.

#### 2.2.5. UPLC Analysis Pretreatment

The pretreatment of crude toxin was as follows. The frozen PDA semisolid medium sample in 2.2.1 and 2.2.2 was melted at room temperature, and 2.0 g of sample was put into a 10 mL tube. Then, the sample was added into 5 mL of CH_3_OH, vortexed for 1 min, and soaked in the dark for 60 min. After centrifugation (7000× *g*, 10 min), the supernatant was dried by N_2_ blowing at 40 ± 0.5 °C. Finally, the sample was added into 1 mL CH_3_OH, vortexed to dissolve, and filtered by a 0.22 µm organic filter membrane for UPLC analysis.

The pretreatment of a food matrix to extract bongkrekic acid was optimized according to the standard of GB5009.189-2016. Specifically, 4.0 g of sample in 50 mL tubes was added into 36 mL CH_3_OH dissolved 1% NH_4_·H_2_O and mixed well. The sample was soaked in the dark for 60 min and extracted in an ultrasound for 30 min. After filtration using filter paper, the filtrate was concentrated to about 3.0 mL in a water bath at 80 ± 0.5 °C. Next, the concentrated sample was transferred to a solid phase extraction column (Waters Corporation, Milford, MA, USA) according to product manual. The column was eluted with 6.0 mL CH_3_OH with 2% CH_3_COOH; the eluate was collected and dried with N_2_ at 40 ± 0.5 °C. Finally, it was dissolved by 1.0 mL CH_3_OH under vortex and filtered by a 0.22 µm organic filter membrane for UPLC analysis.

Sample pretreatment for toxoflavin extraction was modified from [11] and was as follows. Four grams of sample was added into 36.0 mL CH_3_OH with 1% CH_3_COOH. Then, it was soaked in the dark for 60 min, ultrasound treated for 30 min, and filtrated with filter paper. The obtained filtrate was concentrated to about 3.0 mL by a rotary evaporation. Next, the concentrated sample was transferred into a solid phase extraction column (Agilent, Bond Elut Plexa PCX, Santa Clara, CA, USA) according to the product manual. Then, the column was eluted with 6.0 mL CH_3_OH with 5% NH_4_·H_2_O. The eluate was collected and dried by N_2_ at 40 ± 0.5 °C. Finally, the dried sample was added into 1.0 mL CH_3_OH, vortexed to dissolve, and filtered with 0.22 µm organic membrane for UPLC analysis.

#### 2.2.6. UPLC Determination of Toxins

An ultraperformance liquid chromatography (Shimadzu, LC2040C, Kyoto, Japan) equipped with a C18 column (Hypersil GOLD™, 100 mm × 2.1 mm × 1.9 µm) was used for analysis. The column temperature of 30 °C, flow rate of 0.1 mL/min, injection volume of 2 µL, and detection wavelength of 267 nm were used for bongkrekic acid detection. The mobile phase (75:25) consisted of CH_3_OH (A) and acetic acid water (B) at pH of 2.5. Different from the former, the mobile phase of toxoflavin determination consisted of CH_3_OH (A) and 0.1% formic acid water (B) with the ratio of 10:90, and the wavelength was set to 258 nm. Bongkrekic acid (Anpel, Shanghai, China) and toxoflavin (Yuanye, Shanghai, China) standard solution series were used.

### 2.3. Multilocus Sequence Typing of Isolates

The molecular typing method of multilocus sequence typing (MLST) was partially optimized according to the method of the pubMLST database [17]. Specifically, seven housekeeping genes—atpD, gltB, gyrB, lepA, phaC, recA, and trpB—were selected for PCR amplification and sequencing. The PCR reaction system is 0.5 µL of Q5 High-Fidelity DNA Polymerase, 10 µL of Q5 Buffer (5×), 2 µL of dNTP (10 mM), 2 µL of primer F (10 uM), 2 µL of primer R (10 uM), 1 µL of Genomic DNA, and 32.5 µL of ddH_2_O.

The PCR program was as follows. Initial denaturation of 30 s at 98 °C, and 10 s at 98 °C, 20 s at 53 °C, 30 s at 72 °C for 5 cycles, then 10 s at 98 °C, 20 s at 58 °C, 3 min at 72 °C, 5 min at 72 °C, 1 min at 12 °C for 30 cycles. PCR products were sent for sequencing (Aokedingsheng co., Ltd., Beijing, China); then, the sequences were submitted to the PubMLST database for a comparison and determination of the strain sequence types (STs). A neighborhood joining phylogenetic tree was constructed by placing housekeeping genes in tandem by employing MEGA software (version 11.0) with the bootstrap value of 1000 and the Chiplot online tool [25]. PHYLOViZ (version 2.0) software with geoBURST algorithm was used to analyze the relationship between STs and database sequences (See Supplementary Table S2). The goeBURST algorithm, based on triple-locus variant (TLV) hierarchical rules, identified mutually exclusive groups named clonal complexes (CCs) of related STs in the population.

### 2.4. BP Neural Network Prediction of Toxigenic Phenotype

The phenotype prediction model was trained by a back-propagation neural network with two-layer structure, one is sigmoid hidden neurons and the other layer is softmax output neurons (MATLAB, R2022a). The number of hidden neurons was 15 since it generated highest the model accuracy comparing with others. As the input data was MLST base sequences, the expression of which contains four forms [gap, A, T, G, C], thus the input layer is set as a [0, 1, 2, 3, 4] matrix, which corresponds to the base sequence [gap, A, T, G, C] of tandem housekeeping genes respectively. The input layer has 2811 features because of the length of tandem housekeeping genes connected in series is 2811 bp. The output [1, 0, 0, 0] [0, 1, 0, 0] [0, 0, 1, 0] and [0, 0, 0, 1] respectively corresponded to the four toxigenic phenotypes of strains, namely bongkrekic acid negative and toxoflavin negative, bongkrekic acid positive and toxoflavin negative, bongkrekic acid negative and toxoflavin positive, bongkrekic acid positive and toxoflavin positive.

Tandem housekeeping gene sequences and toxigenic phenotypes of 26 isolates, 3 standard strains and 2 referenced strains (BCC1650 and BCC1647, see Supplementary Table S3) were used to train the model. A data enhancement strategy of doubling the housekeeping gene sequences of toxigenic phenotypes of 31 strains was used and 93 observations were provided. These data were randomly divided into three subsets: training set (70%), validation set (15%) and test set (15%). The overall accuracy rate was used to evaluate the accuracy of the model. After repeated optimization and calculation, a well-trained BP neural network prediction model was constructed. Finally, additional 15 referenced strains (See Supplementary Table S3 for details) with known toxigenic phenotypes were used to verify the model.

### 2.5. Data Analysis

The toxin yield differences were compared by one-way ANOVA (GraphPad Prism 8.0.2) and the difference was considered significant when *p* < 0.001.

## 3. Results and Discussion

### 3.1. Strain Isolation and Molecular Identification

In this study, fresh black fungus, soil, fungus bag substrate, field air, irrigation water, drying black fungus, and puddle water were collected. Through the comparison of 16S rDNA sequences similarity with that of *B. gladioli*, we found that 26 among 79 isolates belonged to *B. gladioli* (Supplementary Table S1), given the identity above 97%. Among them, 15 strains were isolated from the black fungus growing period, nine strains were from outdoors drying period, and two strains from the environmental ground rain water, indicating that *B. gladioli* can be detected from the planting, drying process, and production environment of black fungus. Thus *B. gladioli* strains are widely distributed around the whole black fungus production chain.

### 3.2. Toxigenic Phenotyping

**Figure 1:**
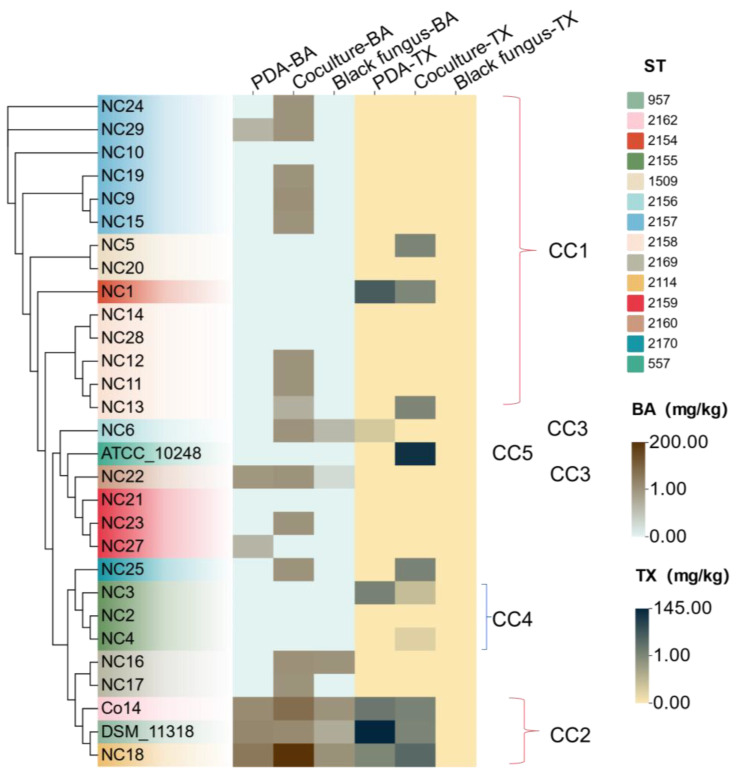
Correspondence of MLST phylogenetic tree based on concatenated sequences of 7 MLST housekeeping genes with a toxigenic phenotype heatmap. The tree was constructed with 26 isolates and 3 standard strains using the neighborhood joining method with a bootstrap value of 1000. The colors of strains were divided according to different STs. The heatmap on the right represents the production of bongkrekic acid (BA) and toxoflavin. CCs at the TLV threshold have been indicated, with CC1-3 being the main and CC4-5 being the minor.

By measuring the toxin yield of 26 isolates and three standard strains cultured separately on PDA medium, *Rhizopus oryzae* co-cultivation PDA medium, and soaked black fungus, we obtained the toxigenic phenotype of the strains and indicated in Figure 1 (the chromatograms can be seen in Supplementary Figure S1). Toxigenic heatmap showed that 20 of the 26 isolates were toxigenic, accounting for 84.6% of the total. Sixteen isolates produced bongkrekic acid and should be classified as *B. gladioli* pv. *cocovenenans* [8]. Since presently no available limit standards on bongkrekic acid in black fungus can be referred to, we take 0.25 mg/kg as the limit according to Chinese national standard GB7096-2014, which stipulates that no more than 0.25 mg/kg of bongkrekic acid can be detected in tremella and related food, considering the similarity of tremella and black fungus as edible mushroom food. Thus, we treated the strains with a toxin amount over 0.25 mg/kg as risk strains, a toxin amount between 0.25 mg/kg and 1.0 mg/kg as medium-risk strains, and a toxin amount more than 1.0 mg/kg as high-risk strains, regardless of culture medium. The results showed that 53.8% (14 strain) were risk strains, including nine medium-risk strains and five high-risk strains, namely NC9, NC16, NC18, NC23, and NC29, producing 1.49–201.86 mg/kg bongkrekic acid. Studies have shown that bongkrekic acid has an oral LD_50_ of 0.68–6.84 mg/kg on mice, and it is fatal for a human to take 1.0–1.5 mg of the toxin orally [14]. It will also cause clinical symptoms such as abdominal pain, vomiting, diarrhea, obesity, irrationality, shock, multiple organ failure, and different cell dysfunction when the intake does not reach the lethal dose [12], which will impact human health seriously. Further, isolates of NC18 showed a much higher toxin-producing yield than other strains. In co-cultivation with *Rhizopus oryzae* on PDA medium, NC18 had a bongkrekic acid and toxoflavin yield of 201.86 mg/L and 45.26 mg/L, respectively. All the evidence above showed that the contamination of *B. gladioli* pv. *cocovenenans* on black fungus is more serious than we thought. The virulent risk strains with high bongkrekic acid yield and their high discovery frequency denote that the food black fungus suffers constant contamination, and the handling of black fungus deserves special scrutiny. Generally, isolates tend to produce more bongkrekic acid on *Rhizopus oryzae* co-cultivation PDA medium than single culture and black fungus. The environmental clue that impacts bongkrekic acid production still needs further research. Since the lack of regularity in toxin production, the prevention of *Burkhoderia gladioli* pv. *cocovenenans* contamination is of paramount significance.

**Figure 2:**
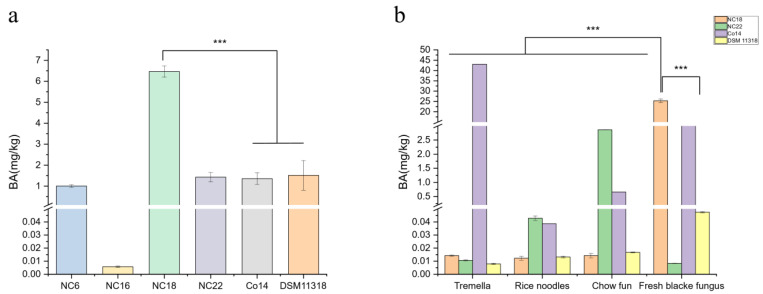
Bongkrekic acid production in soaked black fungus and foods. (**a**) The measurement of four isolated and two standard strains for the production of bongkrekic acid in black fungus soaked for 72 h. (**b**) The measurement of NC18, NC22, Co14, and DSM 11318 for the production of bongkrekic acid in tremella, rice noodles, chow fun, and fresh black fungus foods. *** indicates diferences exist between strains and foods when *p* < 0.001.

It was shown that four isolated and two standard strains produced bongkrekic acid in soaked black fungus after cultivation for 72 h (Figure 2a). Among them, NC18 produced 6.24 mg/kg of bongkrekic acid in soaked black fungus, which was 4–6 times that of a human fatal dose and five times the yield of strain Co14, a food safety incident pathogen leading to four deaths. Thus, NC18 as a representative seriously threatens the table safety of cultivated black fungus. Figure 2b showed that toxigenic isolates could also use other food substrates to produce toxins. NC18 and NC22 produced bongkrekic acid in tremella [26], chow fun, and rice noodles. It was unexpected that the contamination scope of *B. gladioli* pv. *cocovenenans* covers various food items and the result is consistent with the point that the gene of *B. gladioli* has strong plasticity to adapt to different habitats [27].

### 3.3. Multilocus Sequence Typing (MLST) of Isolates

Isolates were subjected to multilocus sequence typing using seven housekeeping genes atpD, gltB, gyrB, lepA, phaC, recA, and trpB. A neighborhood joining phylogenetic tree was constructed by placing housekeeping genes in tandem. The toxigenic phenotype of strains was also illustrated (Figure 1). It was shown that the positions of strains on the phylogenetic tree were correlated with their toxigenic phenotypes. For ST 2157, isolates NC9, NC15, and NC19 were in the same branch, and all of them only produced bongkrekic acid on the co-cultivation medium with *Rhizopus oryzae*. For ST 2158, NC11, NC12, and NC13 were stimulated by *Rhizopus oryzae* to produce bongkrekic acid; thus, they belong to the same branch, while NC14 and NC28 did not produce toxin and belonged to another branch. In addition, NC18, Co14, and DSM 11318, with the same toxigenic phenotype and diverse STs, got together. The three strains produced 0.15–201.86 mg/kg of bongkrekic acid on PDA, *Rhizopus oryzae* co-cultivation medium and black fungus, while abundant toxoflavin (1.6–144.45 mg/kg) can be produced only on PDA and co-cultivation medium, not in black fungus. Above evidence proved the effectiveness of MLST method in identification of toxigenic *B. gladioli* pv. *cocovenenans*.

**Figure 3:**
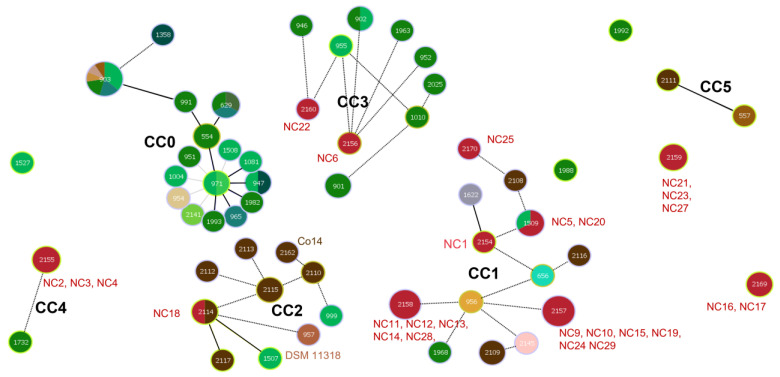
The TLV level geoBURST analysis. The red dots represent strains from black fungus, the green dots represent environmental sources, and the yellow-brown dots represent sources of CF patients (Supplementary Files for details). The solid lines represent only one allele difference and the dotted lines represent two or three allele differences. The distance between STs has nothing to do with the length of the lines.

A visual geoBURST analysis was constructed between the STs of isolates and other strains from diverse sources at the level of TLV. In Figure 3, the distribution of STs in different clone complexes (CCs) reflects the correlation between genetic distance and evolutionary relationship between close relatives of STs [28]. At the threshold of TLV, isolates and standard strain STs were divided into three main CCs, two secondary CCs, and several singletons. Among the three main CCs, the isolated strains were related to other strains including environment and a CF patient source. Therefore, it can be considered that the source of these wild-type isolates may be the environment or human beings; a wild range of routes *B. gladioli* pv. *cocovenenans* may take to contaminate black fungus is inferred. Notably, CC2 contained NC18, standard strains Co14, and DSM11318; thus, this CC needs special care due to high toxicity. The gathering of these strains denotes the effectiveness of MLST for recognition of highly risky strains. The strains from the environment and CF patient all have been reported to be bongkrekic acid positive [29].

In the ecological system, *B. gladioli* are pathogens of some plants. It has been demonstrated that the toxin biosynthetic system plays an important role in enhancing the virulence of *B. gladioli* [27]. In addition, *B. gladioli* strains also have a mutually beneficial symbiotic relationship with the host through their capacities of nitrogen fixation, inhibiting the growth of plant pathogens and protecting insect larvae from natural enemies. In a symbiotic system with insect larvae, *B. gladioli* strains protect insect larvae from infringement by producing secondary metabolites through the regulation of toxin synthesis genes [24]. We inferred that insects may be one of the transmission carriers of *B. gladioli* pv. *cocovenenans* contamination on black fungus, since we observed a large number of insects at the sampling area.

MLST is a general method to classify pathogenic bacteria based on the selected housekeeping gene sequences polymorphism and it is effective in identifying *B. gladioli* and shows important insights into the population dynamics, diversity, and recombination events in this group [30,31]. In this study, the toxigenic phenotype of *B. gladioli* pv. *cocovenenans* isolates was supported by the evolution of housekeeping genes. We found that black-fungus-originating *B. gladioli* pv. *cocovenenans* that carried bongkrekic acid and toxoflavin biosynthesis gene clusters had close evolutionary relationships with strains from different sources such as human beings and the environment. In modern taxonomy, *B. gladioli* includes four subspecies, *B. gladioli* pv. *agaricicola*, *B. gladioli* pv. *allicola*, *B. gladioli* pv. *gladioli*, and *B. gladioli* pv. *cocovenenans*. The first three subspecies exist as plant pathogens, which can cause bulb rot of gladiolus, soft rot of mushroom, root rot of onion, and ear blight of rice, respectively [8]. Currently, *B. gladioli* pv. *cocovenenans* is the only pathovar containing bongkrekic-acid-producing genes as suggested by researchers, and thus they are treated as an independent pathovar. Separately, toxoflavin-producing capability is shared by *B. gladioli*, while *B. glumae*, another species in *Burkholderia* genus, can also secrete toxoflavin [29]. Hence, we think that toxigenic gene cluster of the bongkrekic acid was derived from horizontal gene transfer (HGT) and toxoflavin was from vertical inheritance in *B. gladioli*. Another source of toxin biosynthesis gene cluster may be homologous recombination of genes. It has been found that there may be a specific genome recombination event in the bongkrekic acid pathogenic gene region [32]. Therefore, we came to the conclusion that the origin and evolutionary pathway of biosynthesis gene clusters of bongkrekic acid and toxoflavin are diverse and need further research.

Black fungus infection with *B. gladioli* pv. *cocovenenans* may also be related to fungi and the interaction mechanism between them is more complicated. On the one hand, a symbiont between fungi and the bacteria was formed and secondary metabolites exist as chemical signal driving bacterial-fungal interactions [33]. It is consistent with the fact that some isolates did not produce toxin under single culture condition but secreted bongkrekic acid or toxoflavin on *Rhizopus oryzae* co-cultivation medium. On the other hand, *B. gladioli* pv. *cocovenenans* can take fungi as food, and such function is realized by its T3SS secretion system, which enhances the phagocytosis of bacteria to fungal mycelium [34]. And it has been proved that bongkrekic acid and toxoflavin could inhibit the growth of fungi and their secondary metabolites synthesis [29]. Thus, there was no doubt that black fungus, provides abundant nutrients and competitive motivation for the growth and reproduction of *B. gladioli* pv. *cocovenenans* and its secondary metabolites synthesis. In total, the paths of *B. gladioli* pv. *cocovenenans* to black fungus is complicated and needs to be further explored. Above all, the fact that black fungus is frequently infested by *B. gladioli* pv. *cocovenenans* is confirmed in this study and the ensuing the food safety problems were serious threats to black fungus industry and the table safety of edible black fungus.

### 3.4. BP Neural Network Prediction of Toxigenic Phenotype

**Figure 4:**
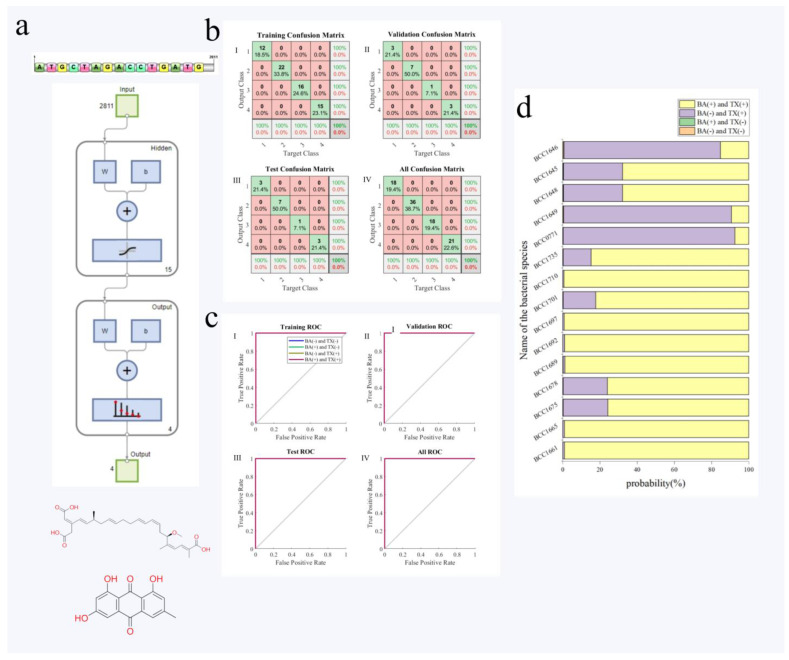
BP neural network prediction of toxigenic phenotype. (**a**) The BP neural network model with the two-layer structure of 2811−15−4; (**b**) the classification accuracy of the training set (I), validation set (II), test set (III) and overall data set (IV) of this model; (**c**) the ROC curve of the model on training set (I), validation set (II), test set (III) and overall data (IV), respectively; (**d**) external data validation results, (+) is toxin positive and (−) is toxin negative, the length of the color block serves as an indicator of the probabilistic assessment for each classification.

A BP neural network model with the biological structure of 2811-15-4 was constructed in this study after repeated optimization and calculation (Figure 4a). Figure 4b shows the classification accuracy of the training set, validation set, test set and overall data set of this model. As can be seen from the figures, the accuracy of the training set, validation set, test set and overall data set was 100%. At the same time, Figure 4c shows the ROC curve of the model, it denotes that the sensitivity and specificity of the training set, validation set, test set and overall data set are all 100%, indicating that the performance of this network was satisfactory.

External data were used to verify the established BP neural network model to evaluate the generalization of the network model. Previous studies have shown that *B. gladioli* species generally has the ability to produce toxoflavin, while the production of bongkrekic acid is unique to *B. gladioli* pv. *cocovenenans* [29]. Therefore, 15 strains with known housekeeping gene sequences from the public database were used to predict the model (Supplementary Table S3). All of these 15 strains can produce toxoflavin, some of which can also produce bongkrekic acid, and all these strains did not participate in the model training at all. The result was shown in Figure 4d with the overall accuracy on external data was 86.7%. Among them, 10 strains produced both bongkrekic acid and toxoflavin were all predicted correctly with a probability of more than 75.7% and 3 strains only produced toxoflavin were correctly predicted with a probability of more than 84.8%. Only two strains BCC1645 with BCC1648 were misjudged as producing bongkrekic acid and toxoflavin with a probability of 67.8%. In general, the accuracy in external data indicated satisfying generalization of the established predict model. This denotes that the model is promising in predicting toxigenicity in new wild isolates, which is valuable in hazardous pathogen detection and offers an convenient method without tedious cultivation and toxin determination.

BP neural network is a kind of feed-forward neural network, which is trained by error back propagation. In the process of training, the input data is processed by the hidden layer to generate the predicted value. By constantly adjusting the connection weights between different layers, the error between the predicted value and the expected value is continuously reduced, and finally a well-trained BP neural network prediction model can accurately reflect the nonlinear relationship between input and output [35]. Usually, evaluating the toxin-producing capability of a strain needs complicated physical and chemical experiments. In this study, A BP neural network prediction model was constructed to predict the toxin-producing capability of the B. gladioli strain based on the relationship between MLST housekeeping gene sequences and toxigenic phenotypes. Comparing with traditional food-borne pathogens detection methods, this model need not to carry out complex and time-consuming physical and chemical experiments. The suspecting *B. gladioli* strains can be quickly and accurately classified according to the toxigenic phenotype. One can simply input housekeeping gene sequences into the BP neural network and the result of whether the strain produce bongkrekic acid and toxoflavin or not is generated. Moreover, with the advent of big-data era and deep sequencing of microbiome, as well as cost reduction on sequencing, the availability of enormous biological genome data contributes greatly to sequence based analysis. The proposed machine learning integrated with multilocus sequencing typing data is rapid, low cost and convenient. It provides an effective method for detecting strain toxigenicity of *B. gladioli* for food safety concerns and in preventive practices.

## 4. Conclusions

In this study, pathogenic strain *B. gladioli* pv. *cocovenenans* was isolated from black fungus for the first time. The blank of extensive evaluation on toxigenic capability of *B. gladioli* pv. *cocovenenans* was filled through studying the toxigenic phenotype of isolated strains in culture medium and various food substrates including black fungus. We realize that *B. gladioli* pv. *cocovenenans* is a serious threat to the black fungus industry and table safety. At the same time, we found that there was a close relationship between the toxigenic phenotype of *B. gladioli* strain with its MLST housekeeping gene sequences. Thus, we constructed a BP neural network model to predict the toxigenic phenotype of *B. gladioli* strains based on MLST sequences and provides a novel method for identifying *B. gladioli* pv. *cocovenenans*. Finally, we realized that black fungus, *B. gladioli* pv. cocovenenans and their environment constitute a mysterious world, waiting for further exploration, and the environmental clues that induce toxin production were still unclear, and the regularity of toxin production by *B. gladioli* pv. *cocovenenans* on different food substrates needs further study.

## Data Availability

Sequence data that support the findings of this study have been deposited in National Center for Biotechnology Information data base and public databases for molecular typing and microbial genome diversity, and the accession numbers are provided within the Supplementary material.

## Supplementary Materials

The following supporting information can be downloaded at: https://www.mdpi.com/article/10.3390/foods13020351/s1, Figure S1: The ultraperformance liquid chromatograms of bongkrekic acid and toxoflavin. Bongkrekic acid were in a and toxoflavin were in b, the purple was standard solution at 5 mg/L and the green were toxins produced by NC18; Table S1: Bacterial species identified based on 16S rRNA gene and its acquired GeneBank accession numbers; Table S2: Reference STs in geoBURST analysis; Table S3: Reference strains in BP neural network prediction model.
