# Supplementary material for: Identification of Burkholderia gladioli pv. cocovenenans in Black Fungus and Efficient Recognition of Bongkrekic Acid and Toxoflavin Producing Phenotype by Back Propagation Neural Network
Source: Foods. 2024 Jan 22;13(2):351. doi: 10.3390/foods13020351 (PMC10815087; doi:10.3390/foods13020351)
Supplement: Supplementary file 1 [file foods-13-00351-s001.zip › foods-2805781-supplementary.pdf]

Table S1 Bacterial species identified based on 16S rRNA gene and its acquired GeneBank accession numbers.

Strain	Sample	Organism	Per. Identy	GenBank
NC1	Black fungus	*Burkholderia gladioli*	97.01%	OP984839.1
NC2	Black fungus	*Burkholderia gladioli*	99.86%	OP984840.1
NC3	Black fungs	*Burkholderia gladioli*	99.58%	OP984841.1
NC4	Black fungus	*Burkholderia gladioli*	99.51%	OP984842.1
NC5	Ground rain water	*Burkholderia gladioli*	99.51%	OP984843.1
NC6	Ground rain water	*Burkholderia gladioli*	99.58%	OP984844.1
NC9	Black fungus	*Burkholderia gladioli*	99.51%	OP984845.1
NC10	Black fungus	*Burkholderia gladioli*	99.51%	OP984846.1
NC11	Black fungus	*Burkholderia gladioli*	99.51%	OP984847.1
NC12	Black fungus	*Burkholderia gladioli*	99.58%	OP984852.1
NC13	Black fungus	*Burkholderia gladioli*	99.58%	OP984853.1
NC14	Black fungus	*Burkholderia gladioli*	99.23%	OP984854.1
NC15	Black fungus	*Burkholderia gladioli*	99.23%	OP984855.1
NC16	Black fungus	*Burkholderia gladioli*	99.65%	OP984848.1
NC17	Black fungus	*Burkholderia gladioli*	99.58%	OP984849.1
NC18	Black fungus	*Burkholderia gladioli*	99.51%	OP984861.1
NC19	Black fungus	*Burkholderia gladioli*	99.72%	OP984851.1
NC20	Sun-curing black fungus	*Burkholderia gladioli*	99.58%	OP984856.1
NC21	Sun-curing black fungus	*Burkholderia gladioli*	99.58%	OP984857.1
NC22	Sun-curing black fungus	*Burkholderia gladioli*	99.30%	OP984858.1
NC23	Sun-curing black fungus	*Burkholderia gladioli*	99.58%	OP984859.1
NC24	Sun-curing black fungus	*Burkholderia gladioli*	98.33%	OP984860.1
NC25	Sun-curing black fungus	*Burkholderia gladioli*	99.58%	OP984861.1
NC27	Sun-curing black fungus	*Burkholderia gladioli*	99.72%	OP984863.1
NC28	Sun-curing black fungus	*Burkholderia gladioli*	99.58%	OP984864.1
NC29	Sun-curing black fungus	*Burkholderia gladioli*	99.44%	OP984865.1

Note that the sequences were submitted and deposited in Genebank in National Center for Biotechnology Information data base(https://www.ncbi.nlm.nih.gov/).

Table S2 Reference STs in geoBURST analysis.

id	isolate	country	source	atpD	gltB	gyrB	recA	lepA	phaC	trpB	ST
916	AU10372	USA	CF patient	223	308	404	254	303	214	261	554
992	3676	Czech Republic	CF patient	223	308	404	254	303	268	261	629
1522	CF120	Germany	CF patient	223	337	492	312	360	269	312	656
1634	AU14120	USA	CF patient	223	309	624	374	360	269	416	901
1635	AU14319	USA	CF patient	223	337	625	375	360	225	417	902
1753	BCC0238	USA	CF patient	223	429	651	387	436	225	428	946
1754	BCC0252	Australia	CF patient	223	308	652	254	303	214	261	947
1760	BCC1620	USA	CF patient	223	432	655	254	303	333	261	951
1761	BCC1621	USA	CF patient	223	308	626	254	303	214	417	903
1765	BCC1645	Australia	environmental	223	308	626	254	303	214	417	903
1766	BCC1646	India	environmental	223	435	655	254	303	334	261	954
1768	BCC1648	USA	environmental	223	308	626	254	303	214	417	903
1769	BCC1649	Zimbabwe	environmental	223	436	655	312	360	269	312	956
1992	VC17927	Canada	CF patient	223	308	626	254	303	214	417	903
1995	VC17964	Canada	CF patient	223	308	626	254	303	214	417	903
1996	VC17963	Canada	CF patient	223	308	655	254	303	214	261	971
2010	VC18110	Canada	CF patient	223	308	652	254	303	214	261	947
2011	VC18109	Canada	CF patient	223	308	626	254	303	214	417	903
2024	VC18242	Canada	CF patient	223	454	655	254	303	214	261	1081
2029	GIMC4595:Bgd-sp17P3	Russia	CF patient	223	308	626	254	303	214	417	903
2451	R-16098	Belgium	CF patient	223	308	655	254	303	214	261	971
2468	QLD127	Australia	CF patient	433	308	626	502	303	214	417	1358

2780	VC17613	Canada	CF patient	365	440	654	392	449	335	432	999
2781	VC17964	Canada	CF patient	223	308	626	254	303	214	417	903
2782	VC18208	Canada	CF patient	365	437	654	391	439	335	433	1507
2783	VC18990	Canada	CF patient	223	309	657	390	360	225	428	955
2784	VC19126	Canada	CF patient	223	337	625	375	360	225	417	902
2785	VC17517	Canada	CF patient	223	308	664	254	303	334	261	1508
2786	VC17878	Canada	CF patient	372	308	670	254	303	214	261	1004
2787	VC18700	Canada	CF patient	223	437	492	312	445	269	312	1509
2789	VC19143	Canada	CF patient	506	440	986	390	624	225	612	1527
2885	SCCH17:Bgd823231	Russia	CF patient	223	308	626	254	303	214	417	903
2886	SCCH11:Bgd82340	Russia	CF patient	223	308	404	254	303	268	261	629
2887	GIMC4605:Bgd-sp12R21	Russia	CF patient	223	308	660	254	303	214	261	965
3114	PP-BB2452	Pakistan	hospital environment	223	437	1061	312	360	484	312	1622
3563	R-10361	USA	CF patient	223	431	1120	396	440	269	312	1732
3571	R-23387	The Netherlands	environmental	223	308	626	254	303	214	417	903
4292	AU0368	USA	CF patient	223	337	665	749	440	225	430	1963
4299	AU3366	USA	CF patient	223	436	417	312	360	269	435	1968
4337	AU35178	USA	CF patient	223	308	404	254	303	268	261	629
4341	AU35657	USA	CF patient	223	885	655	254	303	214	261	1982
4355	AU38680	USA	CF patient	223	309	665	390	360	269	428	1010
4360	AU39558	USA	CF patient	223	337	1270	388	624	269	428	1988
4362	AU39681	USA	CF patient	223	433	624	388	360	225	430	952
4370	AU40190	USA	CF patient	223	308	404	254	303	214	261	554
4373	AU40524	USA	CF patient	223	308	404	254	303	214	417	991

4376	AU41704	USA	CF patient	223	890	1273	388	360	653	209	1992
4377	AU41714	USA	CF patient	223	308	626	254	303	214	417	903
4378	AU41816	USA	CF patient	223	437	655	254	303	214	261	1993
4379	AU42016	USA	CF patient	223	309	624	374	360	269	416	901
4447	AU35053	USA	CF patient	223	437	668	387	360	269	428	2025
4606	BGC01	China	environmental	223	437	492	388	441	269	417	2108
4607	BGC02	China	environmental	223	931	679	312	441	269	417	2109
4608	BGC03	China	environmental	365	440	677	392	439	335	433	2110
4609	BGC04	China	environmental	223	308	417	265	318	225	312	2111
4610	BGC05	China	environmental	365	440	1318	780	884	335	433	2112
4611	BGC06	China	environmental	365	932	1061	779	439	225	433	2113
4612	BGC07	China	environmental	365	437	659	391	439	335	433	2114
4613	BGC08	China	environmental	365	440	1061	779	439	335	433	2115
4614	BGC09	China	environmental	223	431	492	265	360	269	209	2116
4615	BGC10	China	environmental	223	931	679	312	441	269	417	2109
4616	BGC11	China	environmental	365	440	1061	779	439	335	433	2115
4617	BGC12	China	environmental	365	437	624	391	439	335	433	2117
4651	SCCH61:Bgd92-3601	Russia [Europe]	CF patient	223	308	664	254	893	214	261	2141
4656	IH241	Chile	non-CF patient	223	931	655	312	318	269	312	2145

Note that the sequences were submitted and deposited in Public Databases for Molecular Typing and Microbial Genome Diversity (https://pubmlst.org/).

Table S3 Reference strains in BP neural network prediction model.

Strain name	Strain Details	Accession (PacBio)	MLST sequence type	Bongkrekic acid	Toxflavin
BCC1650	*B. gladioli* pv. *cocovenenans*, Indonesia	ERS784828	957	+	+
BCC1661	CF Isolate, USA	ERS784861	959	+	+
BCC1665	CF Isolate, USA	ERS784925	963	+	+
BCC1675	CF Isolate, USA	ERS785062	970	+	+
BCC1678	CF Isolate, USA	ERS784880	973	+	+
BCC1689	CF Isolate, USA	ERS784865	993	+	+
BCC1692	CF Isolate, USA	ERS784914	983	+	+
BCC1697	CF Isolate, USA	ERS784984	989	+	+
BCC1701	CF Isolate, USA	ERS784867	993	+	+
BCC1710	CF Isolate, USA	ERS785039	999	+	+
BCC1735	CF Isolate, USA	ERS785008	1019	+	+
BCC1647	*B. gladioli* pv. *gladioli*, LMG 6882, USA	ERS784943	955	-	+
BCC0771	*B. gladioli* pv. *gladioli*, LMG 2216	ERS784806	557	-	+
BCC1649	*B. gladioli* pv. *gladioli*, LMG 6880t4, Zimbabwe	ERS784812	956	-	+
BCC1648	*B. gladioli* pv. *alliicola*, LMG 2121, USA	ERS784957	903	-	+
BCC1645	*B. gladioli* pv. *alliicola*, LMG 6954, Australia	ERS784911	903	-	+
BCC1646	*B. gladioli* pv. *alliicola*, LMG 6878, India	ERS784928	954	-	+

Note that the sequences used were searched from Public Databases for Molecular Typing and Microbial Genome Diversity (https://pubmlst.org/).

a

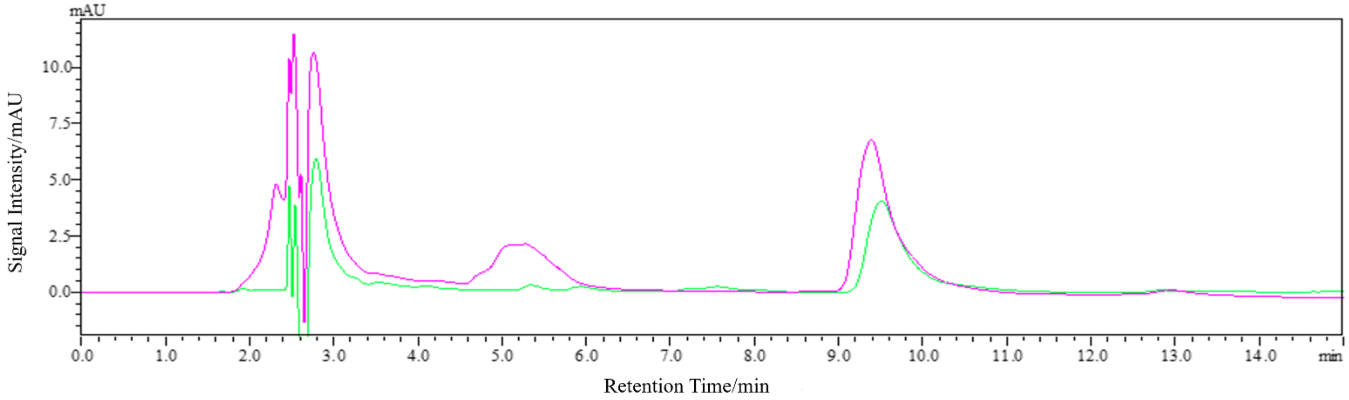

b

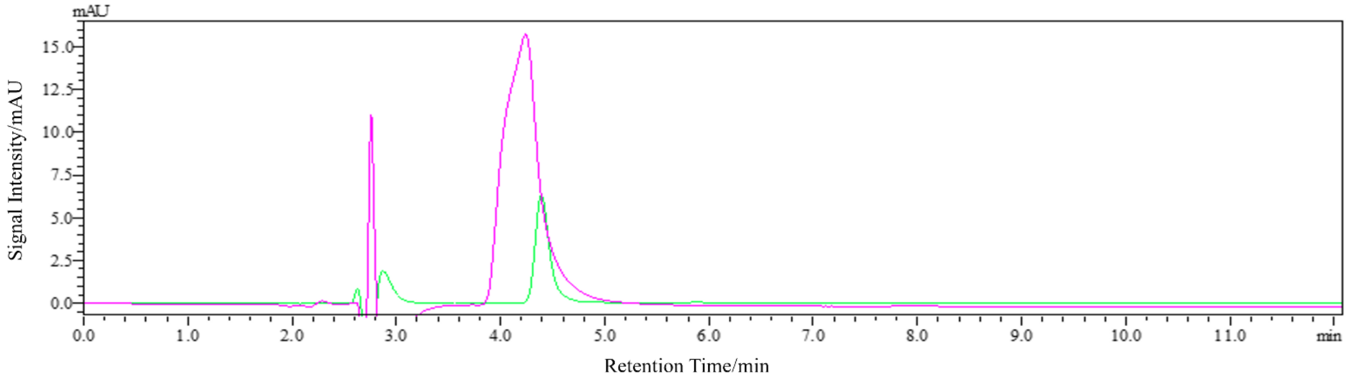

Figure S1 The ultraperformance liquid chromatograms of bongkrekic acid and toxoflavin. Bongkrekic acid were in a and toxoflavin were in b, the purple was standard solution at 5 mg/L and the green were toxins produced by NC18.
